# Preference for using a variety of future HIV pre‐exposure prophylaxis products among men who have sex with men in three US cities

**DOI:** 10.1002/jia2.25664

**Published:** 2021-01-22

**Authors:** Gordon Mansergh, Krishna Kiran Kota, Rob Stephenson, Sabina Hirshfield, Patrick Sullivan

**Affiliations:** ^1^ Division of HIV/AIDS Prevention Centers for Disease Control and Prevention Atlanta GA USA; ^2^ Oak Ridge Institute for Science and Education Oak Ridge TN USA; ^3^ University of Michigan Ann Arbor MI USA; ^4^ SUNY Downstate Health Sciences University Brooklyn NYC USA; ^5^ Emory University Atlanta GA USA

**Keywords:** PrEP, prophylaxis, HIV, MSM, men who have sex with men, prevention products, prevention methods

## Abstract

**Background:**

Daily oral pre‐exposure prophylaxis (PrEP) is available and recommended for men who have sex with men (MSM) at risk for HIV infection. Other HIV prevention products are being developed, including long‐acting injectable (LAI) and event‐based oral and topical formulations. Understanding preferences for potential products by MSM can help direct further development of prevention messaging.

**Methods:**

We present baseline data from HIV‐negative participants enrolled in the US Mobile Messaging for Men (M‐cubed) Study. Participants were asked their likelihood of and rank order preference for using daily oral PrEP and various potential prevention products (one‐ to ‐three‐month injections, 2‐1‐1 sexual event oral dosing, anal or penile gel, or anal suppository), and their sociodemographic characteristics. Bivariate and multivariable logistics regression assessed demographic associations with likelihood of use and rank order preference.

**Results:**

Overall, most MSM reported a likelihood of using LAI (74%), sexual event‐based pills (67%) and penile gel (64%). Men who reported recent unprotected (condomless and PrEPless) anal sex most preferred a penile gel formulation (74%), followed closely by LAI and event‐based pills (73% each). Current PrEP users (vs. non‐users) had greater odds of reporting likelihood to use LAI (AOR = 3.29, 95% CI = 2.12 to 5.11), whereas men reporting recent unprotected anal sex had a greater odds of likelihood to use a penile gel (AOR = 1.79, 95% CI = 1.27 to 2.52) and an anal suppository (AOR = 1.48, 95% CI = 1.08 to 2.02). Hispanic/Latino (vs. White) MSM (AOR = 2.29, 95% CI = 1.40 to 3.73) and, marginally, Black MSM (AOR = 1.54, 95% CI = 1.00 to 2.38) had greater odds of reporting likelihood to use penile gel. Similar patterns were found for rank ordering preference of products, including condoms.

**Conclusions:**

Most MSM were interested in using various potential future HIV prevention products, especially LAI. However, two typologies of potential users emerged: men who prefer sexual event‐based methods (condoms, event‐based pill, sexual gels and suppositories) and men who prefer non‐sexual event‐based methods (daily pill, LAI). Men who reported recent unprotected anal sex preferred a penile gel product most, followed closely by sexual event‐based pills and LAI. Racial/ethnic differences were noted as well. These findings on product preferences can help in formulation development and messaging.

## 1 INTRODUCTION

Currently available for use in the United States, daily administered PrEP in pill form is efficacious and recommended to prevent HIV infection in individuals at high risk for HIV [1]. Since approval by the FDA in 2012, PrEP as a public health strategy for MSM has become increasingly successful [2]. Uptake of, and adherence to, a required daily pill regimen can be an obstacle for people who may benefit the most. Black/African American MSM have been found to have lower daily PrEP use or adherence despite being overrepresented in the HIV epidemic due partly to lack of information and support, although more research is needed on racial/ethnic differences [3, 4]. Taking a daily pill has been a reported obstacle for some MSM. In a 2017 study of MSM (and transgender women), 28% of participants preferred a rectal microbicide gel to a daily oral tablet of PrEP [5]. Long‐acting injectable (LAI) forms of PrEP have also been shown to be popular as a potential administrative method [3, 6, 7], and LAI was recently found to be efficacious in preventing HIV infection [8]. The sparse but emerging literature on product preferences speaks to the need to prepare for a host of potential products and varying preferences among MSM, and a particular need for quantitative research in a large and diverse sample of MSM [6, 9, 10]. In this study, we assess self‐reported likelihood of using various HIV prevention products, including a LAI form of PrEP and sexual event‐based pills, gels and anal suppositories among a large and diverse sample of MSM in three US cities.

## 2 METHODS

Data are from the 2018 baseline assessment of the *Mobile Messaging for Men (M‐Cubed) Study* among MSM in Atlanta, Detroit and New York City (Emory University IRB #00087684) [11]. Briefly, HIV‐negative and HIV‐positive MSM were recruited via street intercept, through local venues, and online to participate in a trial testing the efficacy of a sexual health mobile app. Men consented and completed a computer‐based, self‐report baseline survey at the study site in their respective city. We analysed self‐reported HIV‐negative participants (n = 782) for reported likelihood of using, and rank order preference for, potential future HIV prevention products of LAI (every one to three months); a sexual event‐driven pill (2 pills within 24 hours before sex and two pills over two days after sex); a gel applied to the penis before insertive anal sex; a gel inserted into the rectum with an applicator before receptive anal sex; and a suppository inserted into the rectum 30 minutes before receptive anal sex. Ranking of preferences for use also included currently available products, condoms and a daily PrEP pill. Analyses compared likelihood (“how likely are you to use [product] to prevent HIV infection in the future?”) of use (definitely or somewhat likely (1 to 2) vs. less than likely (neutral, somewhat or definitely unlikely, 3 to 5)) of future products by race/ethnicity (Black, Latino, White, other/mixed), age group (18 to 29, 30 to 39, 40 + years), education level (≤ some post‐high school training, four‐year college degree, ≥some graduate education) and city. We also analysed by unprotected (condomless and PrEPless) anal sex in the prior three months and current daily oral PrEP use, given the importance of those behaviours related to likelihood of using future prevention products. Mean rank ordering of product preference (1 to 6, from highest to lowest) was also analysed for demographic differences. Bivariate and multivariable analyses were conducted on demographic variables and whether or not current use of daily PrEP and condomless/PrEPless anal sex (past three months) is associated with the likelihood of using, and preference for use of, other potential HIV prevention products.

## 3 RESULTS

Nearly a third (32%) of the men reported being current PrEP users, and 36% had unprotected (condomless/PrEPless) anal sex within the prior three months (Table 1). About half (52%) of the sample was White, 20% Black, 16% Hispanic/Latino and 12% other or mixed race/ethnicity. Many (45%) of the men were age 18 to 29 years, 31% age 30 to 39 years and 24% age 40 years or older, and represented well the three cities and education levels.

**Table 1:** Likely to use future HIV prevention product among HIV‐negative men in three US cities, 2018 (n = 782)

Characteristic	n (% overall)	Future HIV prevention product									
		Long‐acting injectable		Event‐based pills		Penile gel		Anal gel		Anal suppository	
		n (%)	AOR (95% CI)	n (%)	AOR (95% CI)	n (%)n (%)	AOR (95% CI) AOR (95% CI)	n (%)	AOR (95% CI)	n (%)	AOR (95% CI)
Overall	782 (100)	560/755 (74)	–	501/749 (67)	–	478/752 (64)	–	407/751 (54)	–	319/752 (42)	–
Current daily PrEP use											
Yes	243 (32)	213 (88)*	3.29 (2.12 to 5.11)*	125 (52)*	0.37 (0.27 to 0.52)*	117 (48)*	0.42 (0.30 to 0.59)*	114 (47)*	0.71 (0.51 to 0.97)*	87 (36)*	0.74 (0.53 to 1.03)†
No (ref)	512 (68)	347 (68)		376 (74)		361 (71)		293 (58)		232 (45)	
Unprotected (condomless/PrEPless) anal sex (3mon)											
Yes	284 (36)	206 (73)	1.16 (0.81 to 1.66)	203 (73)*	1.30 (0.92 to 1.84)	211 (74)*	1.79 (1.27 to 2.52)*	167 (59)*	1.22 (0.89 to 1.67)	141 (50)*	1.48 (1.08 to 2.02)*
No (ref)	497 (64)	365 (73)		314 (63)		287 (58)		253 (51)		186 (38)	
Race/ethnicity											
Black	155 (20)	101 (65)*	0.54 (0.34 to 0.84)*	97 (64)	0.87 (0.57 to 1.33)	108 (69)*	1.54 (1.00 to 2.38)†	83 (54)	0.91 (0.61 to 1.36)	71 (46)	1.26 (0.84 to 1.89)
Hispanic/Latino	123 (16)	90 (73)	0.84 (0.49 to 1.41)	88 (73)	2.12 (1.28 to 3.50)*	87 (71)	2.29 (1.40 to 3.73)*	68 (56)	1.31 (0.84 to 2.03)	52 (42)	1.34 (0.86 to 2.07)
Other/mixed	98 (12)	61 (62)	0.46 (0.27 to 0.76)*	73 (74)	1.90 (1.11 to 3.25)*	65 (66)	1.61 (0.98 to 2.65)†	56 (58)	1.39 (0.87 to 2.23)	43 (44)	1.36 (0.85 to 2.17)
White (ref)	405 (52)	319 (79)		259 (64)		238 (59)		213 (53)		161 (40)	
Age group, years											
18 to 29	353 (45)	247 (70)*	1.06 (0.70 to 1.63)	230 (65)	0.93 (0.62 to 1.40)	215 (61)*	0.53 (0.35 to 0.80)*	180 (51)*	0.57 (0.39 to 0.84)*	130 (37)*	0.56 (0.38 to 0.81)*
30 to 39	239 (31)	195 (82)	1.89 (1.17 to 3.07)*	164 (69)	1.17 (0.75 to 1.82)	147 (62)	0.58 (0.37 to 0.91)*	123 (52)	0.62 (0.41 to 0.93)*	103 (43)	0.73 (0.49 to 1.09)
40+ (ref)	189 (24)	129 (68)		123 (66)		136 (72)		117 (63)		94 (50)	
Education level											
≤ Post HS (ref)	280 (36)	192 (69)		188 (68)		181 (65)		160 (58)		119 (43)	
4‐year college	282 (36)	215 (76)	1.16 (0.77 to 1.76)	187 (66)	1.13 (0.76 to 1.69)	182 (65)	1.50 (1.02 to 2.23)*	148 (53)	0.85 (0.59 to 1.22)	117 (41)	1.07 (0.75 to 1.55)
≥ Post college	217 (28)	162 (75)	1.00 (0.63 to 1.58)	140 (65)	1.07 (0.70 to 1.64)	133 (62)	1.28 (0.84 to 1.96)	110 (51)	0.79 (0.53 to 1.16)	89 (41)	1.03 (0.69 to 1.53)
City/MSA											
Atlanta (ref)	276 (35)	201 (73)		192 (70)*		178 (64)		159 (58)		113 (41)	
Detroit	237 (31)	174 (73)	1.08 (0.70 to 1.67)	164 (69)	0.84 (0.56 to 1.26)	160 (68)	1.16 (0.78 to 1.73)	123 (52)	0.79 (0.55 to 1.15)	103 (43)	1.14 (0.79 to 1.66)
New York	268 (34)	196 (73)	0.96 (0.63 to 1.45)	161 (61)	0.57 (0.38 to 0.83)*	160 (60)	0.78 (0.54 to 1.15)	138 (52)	0.80 (0.56 to 1.15)	111 (42)	1.02 (0.71 to 1.46)

Multivariable regression models include all variables listed within column. AOR, adjusted odds ration; CI, confidence interval; HS, high school; MSA, metropolitan statistical area; –, not applicable.

* *p* < 0.05

^†^ *p* < 0.10.

### 3.1 Reported likelihood of using future HIV prevention products

Most men reported that they were likely to use PrEP in the future (Table 1) via injection every one‐ to three months (74%), sexual event‐based pills (67%), a penile (64%) or anal (54%) gel during sex, or an anal suppository before or after sex (42%). Current daily PrEP users were more likely to endorse future use of LAI, compared to men not currently taking daily PrEP (88% vs. 68%, *p* < 0.05). Alternatively, current daily PrEP users (vs. non‐users) were less likely to endorse event‐based pills (52% vs. 74%), penile (48% vs. 71%) or anal (47% vs. 58%) gel or anal suppositories (36% vs. 45%; *p*’s < 0.05). In multivariable analysis, controlling for demographic variables, current PrEP users (vs. non‐users) were more likely to prefer LAI (Adjusted Odds Ratio [AOR] = 3.29, 95% Confidence Interval [CI] = 2.12 to 5.11), and less likely to prefer an event‐based pill (AOR = 0.37, 95% CI = 0.27 to 0.52), penile (AOR = 0.42, 95% CI = 0.30 to 0.59) or anal gel (AOR = 0.71, 95% CI = 0.51 to 0.97), or, marginally (*p* < 0.10), anal suppository (AOR = 0.74, 95% CI = 0.53 to 1.03) formulations.

A penile gel formulation was the most preferred method of PrEP administration among MSM who reported recent unprotected anal sex (condomless and PrEPless anal sex in the prior three months, 74%; Table 1). Compared to men who did not report recent unprotected anal sex, men with recent condomless and PrEPless anal sex reported greater likelihood of using a penile gel (74% vs. 58%), event‐based pills (73% vs. 63%), an anal gel (59% vs. 51%) and anal suppositories (50% vs. 38%; *p*’s < 0.05) to prevent HIV infections in bivariate analysis. However, reporting of unprotected anal sex was associated only with penile gel (AOR = 1.79, 95% CI = 1.27 to 2.52) and anal suppository (AOR = 1.48, 95% CI = 1.08 to 2.02) formulations in multivariable regression analysis. No difference in likelihood of using LAI was found based on recent unprotected anal sex.

Bivariate differences in likelihood of using products were also found by race/ethnicity and age group, but less so by city and education level (Table 1). In multivariate analysis, Black (vs. White) men had lower odds for reporting likelihood of using LAI (AOR = 0.54, 95% CI = 0.34 to 0.84) and marginally greater odds for likelihood of using a penile gel (AOR = 1.54, 95% CI = 1.00 to 2.38). Hispanic/Latino (vs. White) men had greater odds of endorsing event‐based pills (AOR = 2.12, 95% CI = 1.28 to 3.50) or penile gel (AOR = 2.29, 95% CI = 1.40 to 3.73). Other or mixed race/ethnicity (vs. White) men had differences for similar products as those found for both Black and Hispanic/Latino men.

Compared to MSM age 40 + years, men aged 18 to 29 had lower odds of reporting likelihood of using a penile gel (AOR = 0.53, 95% CI = 0.35 to 0.80), anal gel (AOR = 0.57, 95% CI = 0.39 to 0.84) or anal suppository (AOR = 0.56, 95% CI = 0.38 to 0.81) formulation of PrEP. MSM age 30 to 39 (vs. older men) had greater odds of endorsing an injection product (AOR = 1.89, 95% CI = 1.17 to 3.07) and, similar to the younger age group, lower odds of endorsing a penile or anal gel (see Table 1).

### 3.2 Rank order of preferred HIV prevention products

Overall rank order of preference for HIV prevention products with condoms included as a prophylaxis option (Table 2) was (1 = highest, 6 = lowest rank): LAI (Mean [M] = 3.04, Standard Deviation [SD] = 2.30), daily pill (M = 3.45, SD = 2.22), condoms (M = 3.52, SD = 2.58) and event‐based pills (M = 3.82, SD = 2.04), followed more distantly by anal gel (M = 5.33, SD = 1.83) and anal suppository (M = 5.51, SD = 1.79). Current daily PrEP users (vs. non‐users) ranked LAI and daily oral PrEP higher, and condoms, event‐based pills, anal gel and anal suppositories were ranked lower. Men who reported recent unprotected anal sex (vs. other men) ranked event pills and anal suppositories somewhat higher; there were no other ranking differences based on reporting of recent unprotected anal sex.

**Table 2:** Mean rank ordering (1 = highest) of preferred current and future prevention products among MSM in three US cities, 2018 (n = 782)

	LA Injectable	Daily Pill*	Condom*	Event Pills	Anal Gel	Anal Suppository
	Mean (SD)	Mean (SD)	Mean (SD)	Mean (SD)	Mean (SD)	Mean (SD)
Overall	3.04 (2.30)	3.45 (2.22)	3.52 (2.58)	3.82 (2.04)	5.33 (1.83)	5.51 (1.79)
Current daily PrEP use						
Yes	2.08 (1.48)^a^	2.17 (1.33)^a^	4.53 (2.47)^a^	4.07 (1.96)^a^	5.75 (1.54)^a^	5.81 (1.64)^a^
No	3.45 (2.46)	4.07 (2.31)	3.11 (2.51)	3.69 (2.05)	5.13 (1.94)	5.37 (1.85)
Unprotected (condomless/PrEPless) anal sex (3 months)						
Yes	3.05 (2.25)	3.57 (2.30)	3.75 (2.74)	3.62 (2.02)^a^	5.35 (1.80)	5.30 (1.86)^a^
No	3.04 (2.33)	3.38 (2.18)	3.39 (2.47)	3.94 (2.03)	5.32 (1.85)	5.62 (1.75)
Race/ethnicity						
Black	3.54 (2.45)^b^, ^f^	3.34 (2.12)^d^, ^f^	2.83 (2.29)^b^, ^d^	3.60 (1.93)	5.16 (1.77)^b^, ^c^, ^f^	5.20 (1.78)^b^
Hispanic/Latino	2.99 (2.31)	3.64 (2.27)	3.40 (2.57)	3.94 (2.21)	5.15 (1.87)	5.57 (1.82)
White	2.77 (2.21)	3.27 (2.15)	3.81 (2.61)	3.86 (1.99)	5.57 (1.78)	5.65 (1.77)
Other	3.54 (2.66)	4.14 (2.47)	3.51 (2.67)	3.87 (2.16)	4.78 (1.94)	5.27 (1.81)
Age group, years						
18 to 29	3.16 (2.33)^a^, ^c^	3.46 (2.15)^c^, ^†^	3.06 (2.33)^a^, ^b^	3.79 (2.02)	5.39 (1.88)^b^, ^c^	5.68 (1.75)^b^, ^c^
30 to 39	2.51 (2.07)	3.28 (2.23)	3.83 (2.59)	3.86 (1.97)	5.49 (1.74)	5.71 (1.69)
40+	3.52 (2.41)	3.66 (2.34)	3.99 (2.85)	3.84 (2.17)	4.98 (1.83)	4.90 (1.88)
Education level						
≤ Post HS	3.42 (2.41)^a^, ^b^	3.35 (2.21)	3.22 (2.51)^b^	3.95 (2.12)	5.05 (1.87)^a^, ^b^	5.41 (1.74)
4‐year college degree	2.84 (2.17)	3.47 (2.23)	3.57 (2.59)	3.84 (1.93)	5.43 (1.83)	5.56 (1.87)
≥Post college	2.81 (2.24)	3.54 (2.23)	3.85 (2.62)	3.64 (2.05)	5.56 (1.75)	5.59 (1.75)
City/MSA						
Atlanta	3.03 (2.25)	3.40 (2.27)	3.88 (2.70)^a^, ^b^, ^†^	3.83 (2.04)	5.21 (1.86)^b^, ^†^	5.48 (1.81)
Detroit	3.18 (2.37)	3.35 (2.03)	3.19 (2.40)	3.77 (2.07)	5.26 (1.82)	5.65 (1.78)
New York	2.95 (2.29)	3.59 (2.34)	3.45 (2.57)	3.86 (2.00)	5.52 (1.80)	5.41 (1.79)

HS, high school; LA, long acting; MSA, metropolitan statistical area; SD, standard deviation.

^a^ Row 1 vs. 2 within variable significantly different, *p* < 0.05

^b^ row 1 vs. 3 different, *p* < 0.05

^c^ row 2 vs. 3 different, *p* < 0.05

^d^ row 1 vs. 4 different, *p* < 0.05

^e^ row 2 vs. 4 different, *p* < .05

^f^ row 3 vs. 4 different, *p* < 0.05

* Daily pill and condoms are prevention products currently available and recommended for use.

^†^ *p* < .10 for the single immediately preceding test comparison noted.

White MSM ranked LAI higher than Black and other or mixed race/ethnicity men, but not compared to Hispanic/Latino men. Black MSM were more likely to prefer condoms compared to White and other or mixed race/ethnicity men, but not compared to Hispanic/Latino men. Other or mixed race/ethnicity MSM were less likely than Black and White men to prefer PrEP as a daily pill, but again, not compared to Hispanic/Latino men. Men aged 30 to 39 years (vs. younger and older men) were more likely to prefer LAI; men aged 18 to 29 (vs. age 30 to 39 and age 40+) were more likely to prefer condoms and men age 40+ reported a higher ranking of anal gels and suppositories.

## 4 DISCUSSION

MSM in our study were interested in using various potential HIV prevention products in the future. For each of the products assessed (except anal suppositories), most men in our diverse sample said they were likely to use each formulation method when available. However, given that future products will not all arrive on the market simultaneously, products available for selection among users at any given time will continue to change as emerging products become available.

These results suggest there may be two groups of potential product users: ones that prefer sexual event‐based products (e.g. penile gel, anal gels or suppositories, episodic pills), and ones that prefer non‐event‐based products (e.g. daily pill, LAI), also described as “hot” and “cold” methods, respectively, based on emotional state at the time of use [12]. We found that current daily PrEP users were more likely to prefer one‐to‐three‐month LAI than were non‐users of daily PrEP, whereas non‐users (vs. PrEP users) were more likely to prefer episodic pills, penile and anal gels, or anal suppositories. This is similar to studies that found current PrEP users were more likely to use or prefer LAI, due to the inconvenience of taking a pill every day, and also stress and anxiety associated with missing doses [13, 14, 15]. Of utmost importance are men who reported recent unprotected anal sex, who were most interested in a penile gel formulation to prevent HIV infection, consistent with much earlier research on preferences for future HIV prevention gel products among MSM that found event‐based control of a lubricant‐type product may fit well in the behavioral repertoire of many men [16]. Sexual event‐based pills and LAI were also highly preferred by MSM reporting unprotected anal sex. The study findings are congruent with research on differential preference for prevention product characteristics based on negative attitudes toward using condoms (e.g. prefer a method that does not break the mood or reduce physical sensation) [17], and would suggest a potential prevention method demand segmentation for product outreach, training and promotion once future products are available and approved for use. Targeted prevention messaging based on product preference could be beneficial in curtailing HIV transmission among MSM.

We found racial/ethnic and age group differences in future product likelihood of use, but not many education level or geographical differences of note. For Black men, condoms stood out as a preferred prevention product much more than for other men, whereas LAI was the dominant preferred method for Hispanic/Latino and White men. This underscores the need for differential product development and prevention outreach – including widespread dissemination of information about new products when they are available – that will help groups who could benefit the most, such as Black and Hispanic/Latino men. Penile gel was also preferred more so by MSM of color than by White men, so product development in this arena could potentially benefit Black and Hispanic/Latino men more. Similarly, by age group, targeted product development and prevention messaging could benefit certain age groups. Men in their thirties stood out from older and younger men in their preference for LAI application, and men in their forties and older preferred gels or suppositories more so than younger age groups.

Although the sample of MSM was relatively diverse in terms of age and race/ethnicity in three US cities, it represents a convenience sample, and this has limited generalizability. Besides condoms and daily PrEP, the HIV prevention products described to participants are not currently available for use and thus are hypothetical, which has research limitations but is commonly used in the absence of available products [16, 18]. Further, we assessed self‐reported intentions to use the products and not actual use, which are not always consistent with one another. Qualitative research on why MSM prefer various products over others would further advise product development. Nonetheless, findings from this study can help inform future research on MSM HIV prevention products and help preventionists prepare for the availability of additional products in the future.

As research on new products brings the field closer to multiple consumer options for primary HIV prevention, health officials and practitioners must be ready to help maximize prevention value for individuals and populations. Continued research is needed in the field of prevention product development as more options become available, and more specific characteristics of likely products become known.

In summary, although potential future PrEP formulations of injectable, sexual event‐based pills and penile and anal gels or anal suppositories are generally acceptable to most MSM, there are important differences by race/ethnicity, age and especially men reporting recent unprotected anal sex that have implications for successful uptake and persistence of use. Particular attention should be given to formulations, combinations of products and related marketing efforts so that HIV prevention protection is used by MSM who can benefit the most.

## 5 COMPETING INTEREST

No conflicts of interest to report.

## AUTHORS’ CONTRIBUTIONS

GM conducted the analysis and wrote the manuscript, and all co‐authors (KK, RS, SH, PS) commented on and edited earlier versions. PS and GM lead the project, and PS, RS and SH oversaw implementation in study sites. All authors (GM, KK, RS, SH, PS) have read and approved the final manuscript.

## References

[1] Centers for Disease Control and Prevention .HIV surveillance report. 2014;26:1–123 [cited 2019 Jun 28]. Available from: https://www.cdc.gov/hiv/pdf/library/reports/surveillance/cdc‐hiv‐surveillance‐report‐2014‐vol‐26.pdf

[2] Kasaie P , Pennington J , Shah MS , et al. The impact of preexposure prophylaxis among men who have sex with men: an individual‐based model. J Acquir Immune Defic Syndr. 2017;75:175–83. 28498144 10.1097/QAI.0000000000001354 PMC5488295

[3] Elopre L , Ott C , Chapman Lambert C , et al. Missed prevention opportunities: why young, black MSM with recent HIV diagnosis did not access HIV pre‐exposure prophylaxis services. AIDS Behav. 2020 10.1007/s10461-020-02985-0 PMC7858694 32749626

[4] Mannheimer S , Hirsch‐Moverman Y , Franks J , et al. Factors associated with sex‐related pre‐exposure prophylaxis adherence among men who have sex with men in New York City in HPTN 067. J Acquir Immune Defic Syndr. 2019;80(5):551–8. 30865051 10.1097/QAI.0000000000001965 PMC6417801

[5] Carballo‐Diéguez A , Giguere R , Dolezal C , et al. Preference of oral tenofovir disoproxil fumarate/emtricitabine versus rectal tenofovir reduced‐glycerin 1% gel regimens for HIV prevention among cisgender men and transgender women who engage in receptive anal intercourse with men. AIDS Behav. 2017;21:3336–45. 29119473 10.1007/s10461-017-1969-1 PMC5856109

[6] Calder BJ , Schieffer RJ , Bryndza Tfaily E , et al. Qualitative consumer research on acceptance of long‐acting pre‐exposure prophylaxis products among men having sex with men and medical practitioners in the United States. AIDS Res Human Retrovir. 2018;34:849–56. 30229684 10.1089/aid.2018.0214 PMC6204559

[7] Beymer MR , Gildner JL , Holloway IW , Landovitz RJ . Acceptability of injectable and on‐demand pre‐exposure prophylaxis among an online sample of young men who have sex with men in California. LGBT Health. 2018;5:341–9. 30118399 10.1089/lgbt.2017.0244 PMC6145037

[8] Landovitz RJ , Donnell D , Clement M , et al.Pre‐exposure prophylaxis containing long‐acting injectable cabotegravir is safe and highly effective for cisgender men and transgender women who have sex with men. Abstract OAXLB0101. International AIDS Conference, July 2020; San Francisco (Virtual).

[9] Leu CS , Giguere R , Bauermeister JA , et al. Trajectory of use over time of an oral tablet and a rectal gel for HIV prevention among transgender women and men who have sex with men. AIDS Care. 2019;31:379–387. 30318905 10.1080/09540121.2018.1533223 PMC6382555

[10] Greene GJ , Swann G , Fought AJ , et al. Preferences for long‐acting pre‐exposure prophylaxis (PrEP), Daily Oral PrEP, or condoms for HIV prevention among U.S. men who have sex with men. AIDS Behav. 2017;21:1336–49. 27770215 10.1007/s10461-016-1565-9 PMC5380480

[11] Sullivan PS , Zahn RJ , Wiatrek S , et al. HIV prevention via mobile messaging for men who have sex with men (M‐Cubed): protocol for a randomized controlled trial. JMIR Res Protocols. 2019;8:e16439. 10.2196/16439 PMC6884718 31730043

[12] Linnemayr S . HIV prevention through the lens of behavioral economics. J Acquir Immune Defic Syndr. 2015;68(4):e61–3. 10.1097/QAI.0000000000000499 PMC4334696 25559597

[13] Meyers K , Wu Y , Brill A , Sandfort T , Golub SA . To switch or not to switch: Intentions to switch to injectable PrEP among gay and bisexual men with at least twelve months oral PrEP experience. PLoS One. 2018;13:e0200296. 30024903 10.1371/journal.pone.0200296 PMC6053164

[14] John SA , Whitfield THF , Rendina HJ , Parsons JT , Grov C . Will gay and bisexual men taking oral pre‐exposure prophylaxis (PrEP) switch to long‐acting injectable PrEP should it become available? AIDS Behav. 2018;22:1184–9. 28913659 10.1007/s10461-017-1907-2 PMC5851796

[15] Ellison J , van den Berg JJ , Montgomery MC , et al. Next‐generation HIV pre‐exposure prophylaxis preferences among men who have sex with men taking daily oral pre‐exposure prophylaxis. AIDS Patient Care STDs. 2019;33:482–91. 31603712 10.1089/apc.2019.0093 PMC6839421

[16] Marks G , Mansergh G , Crepaz N , Murphy S , Miller LC , Appleby PR . Future prevention options for men who have sex with men: Intention to use a potential microbicide during anal intercourse. AIDS Behav. 2000;4(3):279–87.

[17] Rader M , Marks G , Mansergh G , et al. Preferences about the characteristics of future HIV prevention products among men who have sex with men. AIDS Educ Prev. 2001;13:149–159. 11398959 10.1521/aeap.13.2.149.19735

[18] Schneider KE , Hamilton White R , O'Rourke A , et al. Awareness of and interest in oral pre‐exposure prophylaxis (PrEP) for HIV prevention and interest in hypothetical forms of PrEP among people who inject drugs in rural West Virginia. AIDS Care. 2020;1–8. 10.1080/09540121.2020.1822506 PMC7981279 32951438

